# Using stakeholder input to inform scenario content: an example from physiotherapy

**DOI:** 10.1186/s41077-019-0102-0

**Published:** 2019-12-20

**Authors:** Sandy Edwards, Neil Tuttle

**Affiliations:** School of Allied Health Sciences, Griffith University, Gold Coast, QLD Australia

**Keywords:** Simulated learning environments, Physiotherapy, Scenario selection, Patient-centred simulation

## Abstract

**Background:**

Simulated learning environments (SLEs) are being embraced as effective, though potentially costly tools, by health educators in a variety of contexts. The selection of scenarios, however, can be arbitrary and idiosyncratic.

**Methods:**

We conducted a stakeholder audit to determine priorities for student learning which would inform scenario design. The process consisted of (1) the identification of stakeholders, (2) consultation with stakeholders to identify their priorities, (3) determination of priorities that could be addressed in the SLE being developed, and (4) incorporating these priorities into scenarios.

**Results:**

The identified stakeholders were the funding body, educational institution and discipline, regulatory agency, accreditation agency, external clinical placement providers, employers of new graduates, patients, and learners. Stakeholder input included a combination of surveys, consultation of online resources, and semi-structured interviews. Identified areas where student learning could be improved included (1) all students not having experience of all populations or ‘essential’ conditions, (2) situations where adverse events had occurred, (3) working with people from diverse backgrounds or those with psychosocial issues including those in chronic pain, (4) communication, (5) situation awareness, and (6) ethical issues.

**Conclusions:**

Ten scenarios were developed considering the stakeholder input. Facilitator notes were written to ensure all facilitators addressed the areas that had been identified. Where possible, simulated patients, with diverse backgrounds, were hired to portray roles even though such areas of diversity were not explicitly written into the scenarios. Whilst the example concerns physiotherapy students within Australia, the principles may be applicable across a range of health disciplines.

**Electronic supplementary material:**

The online version of this article (10.1186/s41077-019-0102-0) contains supplementary material, which is available to authorized users.

## Background

Simulated learning environments (SLEs), which have been defined as techniques ‘to replace or amplify real experiences with guided experiences that evoke or replicate substantial aspects of the real world in a fully interactive manner’ [1], are being embraced as an effective, though potentially costly tool, by health educators in a variety of contexts. It has been suggested that the development of learning activities commences from an assessment of needs and progresses through identification of the learners and learning objectives to selection of educational and evaluation methods [2]. For SLEs in the health professions, the order of development has sometimes been reversed assuming the method (simulation) and then considering the learners, learning objectives, and finally the scenario [3].

Many simulation centres exist in response to specific needs such as accreditation requirements [4, 5] or specialised skills training. In allied health professions, SLEs are frequently used to prepare for [6, 7], replace [8], or supplement [9] clinical placement experiences. In addition, the evolution to a ‘patient-focused’ health service creates the ethical drive to increased competency prior to direct patient contact [4, 5]. There is strong evidence, according to the Health Education and Training Institute’s (HETI) recent review [10], that, provided certain conditions are met, simulation can improve core knowledge and skills and replace up to 25% of clinical placement hours.

Within SLEs, scenario design is considered paramount [10, 11] and is one aspect that has a direct connection to achieving aims. Literature discussing scenario design stresses that simulation activities are part of an educational journey and as such, at a minimum, should be developed to integrate with curricular goals and graduate outcomes [10]. It is also recommended [4] to incorporate the National Safety and Quality Health Service [12] into scenario design. Whilst curricular outcomes and patient safety are often considered [5, 13], strategies to determine the educational needs relevant to other stakeholders are infrequently discussed in the literature [11]. We know of one example that has been described where scenario content was informed by the opinions of several levels of staff and students with respect to learning needs and patient care [14]. In general, the selection of scenario content seems to be subjective and often idiosyncratic.

Response to individual and organisational needs has long been part of determining educational priorities for continuous professional development for qualified practitioners [15] and is driven by the belief that practice changes will be the result. A survey assessing education and training requirements of simulation professionals [16] found a clear need for health educators to have increased support in scenario design. Simulation scenario development was identified as a priority activity within their role, yet 53% of educators thought they needed additional training in this task.

With the purpose of providing health educators with potential direction in scenario design, we describe an approach to conducting a stakeholder audit from a broad range of stakeholders to inform not only the general simulation content, but also the detail within individual scenarios. The concept is described in relation to a specific situation—a simulated telemedicine experience for physiotherapy students undergoing a musculoskeletal clinical placement; it is hoped that the concepts will be useful in other disciplines and areas of practice.

The authors were in receipt of a federal grant to provide SLEs for entry-level students including those from physiotherapy with a primary objective of increasing clinical placement capacity and a secondary objective of supporting rural and remote clinical placements. The project was conducted at the Griffith University in Australia between the second semester of first-year and the first semester of the second-year of a two-year Masters of Physiotherapy program with an integrated clinical placement model. Students undertake a total of five 5-week placements, three of which would occur during these two semesters. The curriculum was accredited, so all essential content for graduates to be registered as physiotherapists was already present within the curriculum. The location of the SLE module within the curriculum was predetermined such that the funded SLEs would replace some hours of clinical placement in a musculoskeletal outpatient setting. The rural and remote priority was partially addressed by the SLEs being conducted via teleconference. Students would remain on their placement sites and be connected to simulated patients (SPs) and facilitators who were on campus.

The question that is the main topic of this paper is how to determine the content of the scenarios. An alternative approach to a formal needs analysis, the stakeholder audit has long been used in private industry as well as public enterprises [17] and consists of (1) identification of stakeholders, (2) determination of the stakes of each holder, (3) determination of how well the needs of each stakeholder are being met, and (4) adjustment to better meet the needs [17].

## Methods and results

### Stakeholder audit

For the purpose of this audit, the steps were rephrased slightly to be (1) the identification of stakeholders from a multi-level perspective, (2) consultation with stakeholders to identify their priorities with respect to student learning needs, (3) determination of common or priority needs, and (4) establishing practical means of incorporating these learning needs into scenarios.

#### Identification of stakeholders

The identified stakeholders, stakes, and methods of gathering input are shown in Table 1. As described above, several aspects of the SLEs were determined by constraints of the funding and the location of the module in the curriculum. In other words, needs of some stake holders including the funding body and the logistics of university curriculum had already been considered. It was not considered to be feasible to consult patients on this occasion. The student consultations were with previous cohorts which provided an indication of their impressions of comparable clinical placements without the inclusion of the SLE.

**Table 1:** Stakeholders and methods of consultation

Category	Organisation/resource	Stake	Method of consultation
Funding body	Health Workforce Australia	Government priorities	Grant application and contract determined extent and structure of SLE
Educational body: discipline	School of Allied Health Sciences, Griffith University: curriculum design	Conforms with university and regulatory requirements	Previously determined timing of SLE and place within the curriculum. Overall content needed to reflect the area of practice
Educational institution	Griffith University graduate attributes	Additional attributes not necessarily covered in regulations	Review of online resources: [18]
Regulatory agency	Australian Health Practitioner Regulation Agency (AHPRA)	Protection of the public. Ensure practitioners conform to ethical and practice standards	Review of online resources [19] Including records of complaints and action taken in responses to complaints
Accreditation agency	Australian Physiotherapy Council (APC): accreditation standards	Ensure graduates are of appropriate standard	Review of online resources [20]
Professional indemnity insurance providers	Professional indemnity insurance providers	Minimise claims	Online documents on risk minimisation including types of frequent claims [21, 22]
External clinical placement providers	Clinical educators for physiotherapy at Griffith University	Ensure students receive relevant experiences and perform to appropriate standard	Online survey
Employers of new graduates	Physiotherapists from public and private system	Firsthand experience of the product of education	Semi-structured interviews
Patients	Not consulted	Ultimate ‘consumers’ of physiotherapy services	Unable to achieve due to time and consultation was not covered by ethics approval
Learners	Physiotherapy students	Learners	Debriefing sessions from previous cohorts following the corresponding clinical placements

#### Consultation with stakeholders

Consultations with stakeholders were divided into three main categories—reviewing online resources, an online survey to clinical educators, and semi-structured interviews with students and employers.

##### Online resources

The Australian Physiotherapy Council (APC) threshold statements [20] and the Griffith Graduate Attributes [18] are policy documents available online. Since the physiotherapy program was accredited, it was considered to be compliant with APC priorities. The Griffith University graduate attributes (knowledgeable and skilled, with critical judgement; effective communicators and collaborators; innovative, creative, and entrepreneurial; socially responsible and engaged in their communities; culturally capable when working with First Australians; and effective in culturally diverse and international environments) extend beyond regulatory requirements to include qualities that are desirable in individuals both personally and within the broader community and were also considered to be addressed within the program. Nonetheless, these priorities and attributes were considered as additional influences that could inform decisions on scenario content.

Complaints to the regulatory body (AHPRA) and information from malpractice insurers provided documented examples of when real or perceived failures had occurred in professional practice and conduct. The most common issues identified from AHPRA were related to professional conduct including inappropriate advertising and boundary violations involving personal relationships. Two practice issues were also identified that related to identification of risk factors. First was a recommendation from the coroner related to a case of undetected deep vein thrombosis and the second was related to the risk of injury during unsupervised exercise.

The material from insurance companies related to minimising the risk of malpractice claims had three main aspects. First was ensuring appropriate practice standards, second the need for comprehensive and contemporaneous patient records, and finally appropriate responses to adverse events.

##### Online survey

A link to an online survey was sent to the 13 facilities that hosted orthopaedic inpatient placements to be distributed to all clinical educators within that facility as well as those involved in supervising new graduates. Questions included identifying gaps in student experiences and new graduate skills. There were seven text-response questions and one to indicate the frequency of their ability to provide each of the experiences that have been deemed to be essential (Table 2). The text-based questions included essential or desirable experiences that the students may not always be exposed to in routine clinical placement, complications that the students must be able to identify, safety concerns, and challenging psychosocial considerations. An opportunity for additional suggestions was provided. There were 22 responses.

**Table 2:** Questions in online survey to clinical educators

1. Can you identify any ‘must know’ orthopaedic conditions that students do not experience at your site? Conversely, in your experience, are there gaps in the new graduates’ experience when it comes to common conditions in your context?	
2. Can you identify any skills that are commonly lacking in new graduates or that students consistently need additional support to develop? Particularly note any safety concerns. Please give specific examples of what behaviours or improvements you would like to see.	
3. What challenging psychosocial situations are your students or new graduates exposed to? Are there specific management strategies you could suggest, or skills you would like them to develop?	
4. What complications do you feel the students or new graduates would benefit from ‘experience of’? Please suggest how you feel these complications should be managed.	
5. Are there any concepts you feel students or new graduates need a stronger understanding of? What specifically would you like them to be aware of?	
6. The Queensland Orthopaedic Physiotherapy Network (2013) identified experiences considered essential for orthopaedic clinical placements. How often are you able to provide an adequate level of these experiences for your students?	
7. Please expand on any concerns identified in question 6	
8. We would greatly appreciate any additional comments or suggestions you might have.	

Only four out of the twenty-two respondents indicated that all ‘must know’ conditions were regularly experienced at their site. The most common gaps were spinal surgery, multi-trauma, and experience with patients across the lifespan—that is, students either primarily experienced paediatric patients or had minimal experience with a younger population. For eight of the fifteen items that the Queensland Orthopaedic Network identified as essential experiences, 90% of respondents indicated that they were always able to provide adequate exposure. The four items where less than 80% of the respondents indicated were ‘always covered’ were trauma, including peripheral, spinal, and multiple (35%); across the age continuum (61%); elective surgery, including upper limb, lower limb, and spinal (74%); and experience with slings and splints (78%).

The most common skills that were identified as lacking were (1) skills in patient care, e.g. getting in and out of bed and mobilising (*n* = 13), (2) communication—documentation and handover skills particularly between disciplines (*n* = 8), (3) risk minimisation (*n* = 7), and (4) understanding protocols (*n* = 4). Respondents identified a wide range of challenging psychosocial situations, but none addressed the second part of the question where they could suggest management strategies.

Respondents indicated a wide range of complications that students would benefit from experience of which were mostly related to aspects of psychosocial situations or risk minimisation as mentioned above. The aspects that the respondents felt students or new graduated ‘needed a stronger understanding of’ included clinical reasoning, risk minimisation, working with psychosocial issues, and communication particularly within the multidisciplinary team. No new topics related to scenario content were found in the final open-ended question.

##### Semi-structured interviews

After each clinical placement, students have a debriefing session which included discussion of areas where they would have liked to have been better prepared and experiences which had not been available to all students. In this instance, the information from previous cohorts who did not have access to the SLE was considered. Semi-structured interviews were conducted by one of the authors (SE) with six employers of new graduates including those from private practices and community health services who do not supervise students on orthopaedic inpatient placements. The interviewer knew approximately half of the employers who were interviewed. Student responses on gaps in areas of practice largely mirrored those found in the clinical educator survey. Students reported that they could have been better prepared for record keeping and communication, particularly in relation to their clinical educators.

Employers of new graduates identified communication skills and professional behaviour as the areas that could be improved. Their impression was that attitudes towards learning, willingness to request assistance, and lifelong learning skills were more important than specific clinical skills. A specific area of communication described by employers was in relation to job applications and interviews.

### Identified gaps which could be addressed in a SLE

The two authors collated the information gathered from the stakeholders and presented this to the other two physiotherapy educators who were involved in developing the scenarios. The team reached consensus on which gaps that had been identified could be reasonably addressed within the SLEs that were being developed within the constraints of our funding agreement.

Gaps that were found through the consultative process that could be addressed in an SLE included (1) skills identified as lacking, (2) all students who did not have experience of patients with all populations or those with ‘essential’ conditions, (3) specific situations where adverse events had occurred, (4) working with people from diverse backgrounds or those with psychosocial issues including those in chronic pain, (5) communication including written (case notes and interprofessional communication) and verbal (with patients, educators, and other professionals), (6) situation awareness including how unexpected factors might impact on patient care, and (7) ethical issues including professional behaviour and boundary violations. In addition, although not actual gaps, there were areas where, when possible, content could be selected that was relevant to the Griffith University graduate attributes [18].

Some areas that were found were not considered to be able to be addressed in the current setting include (1) administrative aspects of private practice, (2) job applications and interviews, and (3) conforming with advertising regulations. Each of these areas, however, has subsequently been addressed in other areas of the curriculum.

### Incorporating findings into scenarios

A total of ten scenarios were developed (see Additional file 1). Facilitator notes were written to ensure all facilitators addressed the topics that had been identified. Where possible, SPs with diverse backgrounds were hired to portray roles even though such areas of diversity were not explicitly written into the scenarios.

#### Populations and essential experiences

To meet the desire for student exposure to patients across the lifespan, scenarios were written to include patients across a wide age range. One scenario included a young patient with cerebral palsy. In this instance, we ensured authenticity by having an SP who was, at the outset of the project, a first-year university student with cerebral palsy and was able to portray an early teenage patient. Priorities of cultural competency were responded to by including patients from diverse socio-economic, cultural, and gender identity backgrounds. The exact nature of this diversity was not necessarily written into the scenarios but was a priority for recruitment of SPs for each iteration of the SLE.

#### Specific situations

Two scenarios were developed based on reports from AHPRA of adverse events. One scenario duplicated the situation recounted in the coroner’s report of an undiagnosed DVT. Contrary to the real-life setting, the SLE was structured with enough cues that the students would be expected to recognise the risk and ‘save’ the patient’s life. Debriefing included reference to the real-life situation where the patient had died and discussion of how the risk factors could have been missed. Another SP returned for their second treatment having sustained an injury whilst they were performing their prescribed exercises through no direct fault in their care. Debriefing could then consider risk minimisation strategies as well as the student response to an adverse event.

Patients with multi-trauma, spinal surgery, or in some instances surgery to peripheral joints were identified by clinical educators and students as areas where all students on clinical placement did not necessarily have experience. Scenarios included patients with all of these conditions.

#### Ability to work with patients with psychosocial issues

Working with patients with psychosocial issues is another area which is ideally suited to an SLE due to the safety of the environment and the ability to control the situations. Aspects that were written into the scenarios included patients with (1) two very different responses to chronic pain, (2) depression, (3) substance abuse, and (4) social isolation. Accurate portrayal of psychosocial issues requires clear conceptualisation and descriptions in the written scenario, but also rehearsal and role moderation for each SP. Debriefing strategies were described in the facilitator notes to guide discussion.

#### Communication

Scenarios were specifically designed to demand a variety of communication skills. The choice of a telehealth platform in itself challenged communication skills in a way not demanded in routine practice. Debriefing strategies were selected to encourage reflection [23] which utilised multiple perspectives (peer, facilitator, patient, and self) and were structured to target communication skills. Peers were encouraged to observe for the use of open-ended questions and active listening strategies. Facilitators addressed communications skills during the debriefing process, and time allowing, students were given the opportunity to repeat experiences to solidify their learning. Time permitting, the SPs provided feedback on communication ‘from the patient’s perspective’. Students wrote patient records from their encounters and were then provided with ‘example documents’ for the students to compare and reflect on. In addition, students were given access to videos of their personal performances to reflect on the interactions independently.

A specific area of communication described by employers was in relation to job applications and interviews. Although it was not possible to incorporate job applications into this SLE, a later separate simulation was developed to address this skill [24]. To encourage interprofessional learning, interactions with dietetics, exercise physiologists, psychology services, and general practitioners, in both a responsive and proactive way, were integrated into the scenarios. Representatives from each discipline were consulted to ensure authenticity of scenarios design. For written communications, ‘example documents’ were provided to students after they had made an attempt at the task.

#### Situation awareness

Situation awareness has been described as consisting of three components: perception of elements in the current situation, comprehension of current status, and projection of future status [25]. It therefore does not require multiple events to be occurring simultaneously as is sometimes thought. The consultations in the current simulations were one-on-one and conducted via teleconference so there was little opportunity for multiple events to be occurring simultaneously. Nonetheless, it was essential for the students to be aware of and understand the patients’ situation beyond what was happening in front of them as well as project what the possible outcomes might be. Scenarios were structured to include a range of aspects that would be important to consider including family interactions, eligibility for workers’ compensation, and psychosocial issues. Prompts for the discussion of future implications of the patients’ situation were therefore included in the facilitator’s notes.

#### Ethical issues

SLEs are ideally suited to working with ethical issues due to being able to present controlled situations and due to the safety of the environment. One scenario included a patient who had been affected by a boundary violation from a previous practitioner which would have triggered the need for mandatory reporting to AHPRA by the student. The need for professional behaviour was incorporated into facilitator notes for all scenarios. The pre-briefing for each session included a discussion of simulation being a safe environment and ‘what happens in simulation, stays in simulation’. Fortunately, the one exception that was explicitly stated was professional behaviour. One pair of students exhibited explicitly racist behaviour which they would not accept responsibility for during the debriefing sessions. As a result, the concerns were escalated with the facilitator’s impressions and a video recording of the interaction supplied to the relevant head of school.

## Discussion

A case is explored where a stakeholder audit process was used to inform scenario content. Although a range of priorities was found, there were few conflicting demands from different stakeholders. For example, the students and facilitators identified similar gaps in student experiences; the focus on record keeping was identified by clinical educators, employers, and insurance companies; and the need for situation awareness was implicit in input from the regulatory body, clinical educators, and employers.

For the future, deeper engagement with stakeholder classification literature may refine the identification and prioritisation process. In our context, including new graduates’ perspectives of learning need, from their very recent past, may have given us alternative insights. In addition, our frame of reference was very directed by the physiotherapy profession, a traditional approach to student education. It might be enlightening to seek the opinion of other disciplines who work regularly with our students or new graduates (e.g. nursing staff, doctors, other allied health). Similarly, the patients and their family’s thoughts might challenge us further.

Other learnings included the need to communicate the educational priorities to, at the minimum, the stakeholders immediately affected by the intervention. For example, whilst a priority from our funding was improving service to rural and remote Australia, neither the clinical educators nor the students appreciated the role of the telemedicine platform. As such, subsequent iterations used face-to-face simulation with similar scenarios. That is not to suggest that there is no value in telehealth for simulation, rather that better integration between educational and health technical systems would be necessary to make it more effective in the setting used in the current example.

## Conclusion

A limited stakeholder audit was used to inform the content of simulation scenarios and structure of SLEs. Although the example in this paper was specific to physiotherapy students within Australia, it is hoped that the concepts could be useful across a range of health disciplines including in other countries.

## Additional file

Additional file 1: Summary of scenarios developed from consultation with stakeholders (DOCX 17 kb)

## Data Availability

The datasets used and/or analysed during the current study are available from the corresponding author on reasonable request.

## Abbreviations

AHPRA: Australian Health Practitioner Regulation Agency
APC: Australian Physiotherapy Council
SLE: Simulated learning environments
SP: Simulated patient
