# Supplementary material for: Using stakeholder input to inform scenario content: an example from physiotherapy
Source: Adv Simul (Lond). 2019 Dec 20;4(Suppl 1):20. doi: 10.1186/s41077-019-0102-0 (PMC6923834; doi:10.1186/s41077-019-0102-0)
Supplement: Supplementary file 1 — Summary of scenarios developed from consultation with stakeholders (DOCX 17 kb) [file 41077_2019_102_MOESM1_ESM.docx]

Additional File 1. Summary of scenarios developed from consultation with stakeholders

| Patient & condition | Additional defined characteristics | Primary learning outcome/s | Gaps or attributes addressed* | Stakeholders** | Adjustments to scenario |
| --- | --- | --- | --- | --- | --- |
| 1. Judith:   Calf pain. | Characteristics defined by actual case | Identification of possible deep vein thrombosis (DVT)  Appropriate management once risk identified | C) Coroner’s finding of missed diagnosis resulting in death, recommendation for increased training for health professionals in identification of DVT  E) Ensure adequate case notes and patient understanding of risks | 1)  2), 4), 5) | Specific information provided on risk factors for DVT prior to SLE with the intention that students find the correct diagnosis and ‘saves’ the patient’s life |
| 1. Maree:   Acute low back pain | Socially disadvantaged, single mother  Fear of being fired from work due to injury  Reticent communicator | Management of acute low back pain  Mandatory reporting of boundary violation from previous health professional | B) Exposure to patient with acute low back pain  D) psychosocial and employment issues  G) Boundary violation a source of complaints against PT | 5),  2), 5), 7)  1), 2) | Scenario found to be too complex for students at this level. Boundary violation removed from scenario and included as a ‘what if’ discussion point in debriefing.  Written communication with general practitioner |
| 1. Laura:   Rehabilitation following wrist fracture | Female, elderly  Sensitive to language that promotes fear | Management of fear-avoidance behaviours.  Identification of falls risk. | A), B), Early pattern consistent with development of chronic pain  E) Effective communication necessary to convince that movement and activity are safe  C), F) Recognition of falls risk | 5), 6), 7)  4), 5)  4), 6) | Written communication with general practitioner.  Level of fear avoidance and readiness to change of patient needed to be modulated depending on when in program scenario presented |
| 1. Mick:   Multi-trauma | Lives alone in a caravan park, with an external toilet block. | Ability to prioritise and adjust treatment due to concurrent injuries/disabilities.  Manage non-compliance with medical instruction | B) Patients with multi-trauma not seen by all students  F) Needs of patient in atypical living situation  E) Motivation of non-compliant patient | 5)  2)  2), 5) |  |
| 1. James:   Neck pain | Alcohol abuse, secondary to bereavement, resulting in fall causing injury | Recognise depression  Responsive to patient emotional state (grief: bereavement) | B) Patients with neck pain not seen by all students  E), F) Recognition of and response to depression and alcohol abuse | 2)  2), 5), 6) | Scenario worked well. Added written communicating with general practitioner |
| 1. Helen: Carpal   tunnel syndrome with concurrent neck pain | Exposure to legal requirements of working with patient covered by workers compensation | Recognise and respond to requirements of working with patients covered by workers compensation | B), Condition not seen by all students  E), F) Employers | 5), 6), 7) | Written communication necessary for patient eligible for worker’s compensation |
| 1. Emily: 21 y/o   student. Fall after feeling faint two weeks ago. Internal fixation of fractured head of radius. | Recognise body image issues and inadequate nutrition | Routine management of internal fixation of upper limb. | B) Condition not seen by all students  D), E), F) Impact of perceived need for weight loss on general health and risk factors | 6)  2), 5), 6) | Referral letter to dietitian |
| 1. Sarah: lumbar   discectomy | Nil | Management of patient after spinal surgery | B) Condition not seen by all students | 5). 6) |  |
| 1. Grace: 17 Y/O with Cerebral Palsy and two-month history of knee pain | Concurrent neurological condition | Management of patient with concurrent musculoskeletal and neurological conditions | B) Condition and population not seen by all students | 2). 5), | Patient portrayed by SP with neurological condition. Scenario would be adjusted depending on the neurological conditions present in SPs who were available |
| 1. Suzanne: 45 Y/O   Patella-Femoral pain. | Type 2 Diabetes and history of dizziness. | Management of chronic systemic condition. Differential diagnosis of dizziness | B) Condition not seen by all students  F) Recognition and response to patient with diabetes and dizziness | 5), 6)  2), 5), 6), 7) | Written communication with general practitioner |
| Additional factors introduced by actors with diverse cultural and social backgrounds introduced as available from the pool of actors |  | Ability to communicate with and recognise needs of patients from diverse backgrounds and cultures | A), D), E), F), G) | 2), 5), 6), 7) | Diversity of SPs included age; identity in terms of ethnic, cultural and gender |

* Gaps or attributes:

1. Skills identified as lacking
2. All students did not have experience of patients with all populations or those with ‘essential’ conditions
3. Specific situations where adverse events had occurred
4. Working with people from diverse backgrounds or those with psychosocial issues including those in chronic pain
5. Communication including written (case notes and interprofessional communication) and verbal (with patients, educators and other professionals)
6. Situational awareness including how factors besides their primary condition might impact on patient care
7. Ethical issues including professional behaviour and boundary violations

** Stakeholder

1. Australian Health Practitioner Regulation Agency
2. Griffith Graduate Attributes
3. Australian Physiotherapy Council
4. Professional indemnity insurance providers
5. Clinical placement providers
6. Employers
7. Learners
